# Medical students’ perceived stress and perceptions regarding clinical clerkship during the COVID-19 pandemic

**DOI:** 10.1371/journal.pone.0277059

**Published:** 2022-10-31

**Authors:** Hae Won Kim, Jong Won Hong, Eun Ji Nam, Ka Young Kim, Ji Hye Kim, Jee In Kang

**Affiliations:** 1 Department of Medical Education, Institute of Behavioral Science in Medicine, Yonsei University College of Medicine, Seoul, Republic of Korea; 2 Clinical Clerkship Committee, Yonsei University College of Medicine, Seoul, Republic of Korea; 3 Department of Plastic and Reconstructive Surgery, Institute for Human Tissue Restoration, Yonsei University College of Medicine, Seoul, Republic of Korea; 4 Department of Obstetrics and Gynecology, Institute of Women’s Life Medical Science, Yonsei University College of Medicine, Seoul, Republic of Korea; 5 Office of Educational Affairs, Yonsei University College of Medicine, Seoul, Republic of Korea; 6 Department of Psychiatry, Institute of Behavioral Science in Medicine, Yonsei University College of Medicine, Seoul, Republic of Korea; Georgia Southern University, UNITED STATES

## Abstract

**Background:**

It is important to ensure that both the qualitative and quantitative aspects of clinical education are maintained during the pandemic. Understanding students’ views on clinical rotations and the extent of their perceived pandemic-related stress would thus be useful for designing and implementing effective clerkship programs. Therefore, this study aimed to investigate perceived stress and perceptions regarding clinical clerkship among incoming clinical students (third year) and senior clinical students (fourth year) during the COVID-19 pandemic.

**Methods:**

After completing orientation programs at the beginning of the academic year, we surveyed students on their perceived stress, their general perspectives regarding the appropriate scope of clinical clerkship, and their preferences regarding level of participation in clerkship. We examined the differences in stress and clerkship-related perceptions based on the students’ study year and sex using independent t-test, chi-squared test, and Fisher’s exact test. In addition, the influences of stress, sex, and study year on clerkship-related perceptions were examined using multinomial logistic regression.

**Results:**

The independent t-test indicated that third-year students experienced lower stress than did fourth-year students. Clerkship-related perceptions also differed significantly between third- and fourth-year students. Multinomial logistic regression analyses on the scope of and participation levels in clinical clerkship revealed that third-year students had significantly lower odds of preferring a limited range of clinical rotations and lower engagement in clerkships compared to fourth-year students.

**Conclusion:**

The COVID-19 pandemic has affected clinical education and, consequently, medical students’ inclination toward active participation in clinical rotations. It is thus essential to understand students’ views and provide them with relevant intra-pandemic educational supports.

## Introduction

The coronavirus disease 2019 (COVID-19) outbreak has interrupted and suspended various aspects of medical education and posed certain fundamental challenges in this regard (for example, the transition to online learning and assessment) [1]. Notably, here, clinical education is one of the most affected areas, as students’ direct contact with patients has been minimized in order to maintain the safety of both parties. Although medical schools have struggled to adapt their clinical curriculums to these unprecedented changes (for example, providing virtual clerkship programs) [2], students’ participation in patient care and face-to-face contact with their educators has been limited [3]. This scarcity of in-person education avenues could raise certain concerns from students as well as educators—especially regarding the question of whether medical students are learning enough in order to become competent physicians [4]. A previous study on final year medical students’ perceptions showed that a severe disturbance in terms of assistantships could impact students’ preparedness and confidence, which are necessary characteristics for transitioning to medical practice [5].

Along with academic challenges, varied pandemic-related impacts on personal and social activities have decreased well-being and increased mental health problems among students [6]. Reportedly, physicians and health care professionals have experienced intra-pandemic mental health problems more frequently and seriously than the general population [7, 8]. The factors affecting physicians’ mental health include high infection risks, shortage of protective equipment, heavy workloads, and increased infection risks in their families [9, 10]; some of these issues may also affect clinical phase medical students. Previous studies on medical students’ intra-pandemic stress and mental health have indicated that they often experience a significant level of distress [11, 12]. As clinical phase medical students must fulfill dual responsibilities—as students and members of healthcare teams—their distress may also be greater compared to that of preclinical students.

The clinical phase of medical education forms a critical period for students’ personal and professional development [13]. They often gain experience in various medical disciplines, and their authentic learning occurs through engagement in patient care-related activities [14]. Thus, it is important to ensure that both the qualitative and quantitative aspects of clinical education are maintained during the pandemic. Understanding students’ views on clinical rotations and the extent of their perceived pandemic-related stress would thus be useful for designing and implementing effective clerkship programs. However, few previous studies have examined students’ intra-pandemic perceptions toward clerkship [15, 16].

In South Korea, most medical schools suspended clinical clerkships when the outbreak began in March 2020 [17] but recommenced them within three to four weeks. However, most institutions (including ours) did not allow clinical rotations to be conducted in emergency, intensive care, and isolation units. Thus, the present study aimed to explore clinical phase medical students’ intra-pandemic stress, perceptions regarding clerkship scope, and inclination toward engaging in clinical practice within a medical school context in South Korea. We examined the influences of students’ study year and sex on perceived stress and perceptions regarding clerkship and whether stress, sex, and study year could affect their preferences regarding the depth and range of their clinical education.

## Materials and methods

### Ethics statement

The Institutional Review Board at Severance Hospital, Yonsei University Health System, approved this study as research using data obtained from normal educational practice in commonly accepted educational settings and thus considered to be an IRB-exempt study (IRB no. 4-2021-0351). Therefore, a waiver of informed consent was granted by the Institutional Review Board at Severance Hospital, Yonsei University Health System. All methods were carried out under the declaration of Helsinki, and the entire analysis was reported according to the Strengthening the Reporting of Observational Studies in Epidemiology (STROBE) statement for cross-sectional studies.

### Design, setting, and participants

A cross-sectional observational study involving third- and fourth-year clinical phase medical students was conducted at the Yonsei University College of Medicine, Seoul, South Korea. While the fourth-year students had experienced intra-pandemic core clerkships in the preceding year (2020), the third-year students had no clinical experiences before and were about to enter the clinical phase. Upon completing clerkship orientation programs at the beginning of the academic year, each student group received a survey for assessing their perceived stress and perceptions regarding clinical clerkships. Third-year students completed a paper-based survey between February 5, 2021, and February 9, 2021, while fourth-year students completed a web-based survey between February 27, 2021, and March 2, 2021. These periods corresponded to the cessation of the third wave of the COVID-19 outbreak in South Korea. Survey responses were obtained anonymously, and personally identifiable information was not collected.

### Measures

#### Perceived stress scale

We used the perceived stress scale (PSS) [18] to assess the students’ perceived stress levels regarding the COVID-19 pandemic. As the original version was designed to measure uncontrollable and overloaded feelings over the past month, we included an explicit instruction to indicate such feelings and thoughts related to COVID-19. The PSS contains 10 items, which are rated on a 5-point Likert scale ranging from ‘Never’ (0) to ‘Very often’ (4). The total score ranges from 0 to 40, and higher scores indicate higher levels of perceived stress. The severity levels of stress can be categorized according to the total scores, such as scores ranging from 0–13 as low stress, 14–26 as moderate stress, and 27–40 as high stress [19]. In this study sample, the internal consistency value measured using Cronbach’s alpha was 0.85.

### Scope of clinical clerkship during the pandemic

We asked students how they perceive the appropriate scope of clerkships during the COVID-19 pandemic, and they were prompted to choose from among the following statements:

“Considering the current COVID-19 pandemic situation, what do you think is the appropriate scope of clinical clerkships?”

- Proceed with regular rotation schedule including emergency room (ER) and intensive care unit (ICU).
- Proceed with regular rotation schedule except for ER/ICU/isolation rooms.
- Carry out the clinical rotations in the general ward and outpatient clinic settings in small groups in a limited manner.
- Completely discontinue of all clinical rotations.

As each response option reflects a distinct category, this variable was used as a nominal variable with four categories.

### Level of participation in clerkship during the pandemic

The following questions and statements were used for assessing the students’ intra-pandemic perceptions regarding the appropriate participation level for clerkships:

“Considering the current COVID-19 pandemic situation, what do you think is the appropriate participation level for clinical clerkships?”

- Actively participate in clinical rotations and COVID-19 pandemic-related volunteer work.
- Actively participate in clinical rotations but not in COVID-19 pandemic-related work.
- Participate only in essential clinical rotations for student safety (infection prevention).
- Minimize clinical rotations despite missing out on essential clinical rotations because students’ safety is the highest priority.
- Minimize clinical rotations, as students may be asymptomatic carriers.

As each response option reflects a distinct category, this variable was used as a nominal variable with five categories.

### Statistical analysis

In this study, the descriptive statistics were presented as the mean and standard deviation for continuous variables and frequency and percentage for categorical variables. Independent-samples t-test and one-way analysis of variance were used to compare the students’ perceived stress based on study year, sex, and clerkship-related perception variables. The normality of data was examined with the Shapiro-Wilk test, and there was no violation of the normality assumption for proceeding with the independent samples t-test and analysis of variance. Chi-squared and Fisher’s exact test were used to analyze the associations between categorical variables, such as demographic factors and perceptions of the appropriate scope and participation levels for clinical clerkships. Furthermore, the possibility of the PSS score, sex, and students’ study year predicting clerkship-related perceptions was modeled using multinomial logistic regression. All analyses were conducted using the R software (version 4.1.0; https://cran.r-project.org/), and the internal consistency of the PSS was measured with the R package *psych*. The multinomial logistic regression was performed with the R package *mlogit*. The level of statistical significance was set at α = 0.05.

## Results

### The participants’ characteristics

The response rates for the survey were 97.3% (110/113) for the third-year students and 58.0% (69/119) for the fourth-year students. We did not collect students’ age information; therefore, because of the responses’ anonymity, we could not identify their age after the survey. Regarding sex distribution, there was no difference between third-year and fourth-year students (p = 0.068) (Table 1).

**Table 1:** Demographic characteristics and stress levels of the study participants.

	Third-year students (n = 110)	Fourth-year students (n = 69)	t or χ^2^	*p*-value
Sex, n (%)			χ^2^ = 3.32	0.068
Female	29 (26.4)	28 (40.6)		
Male	81 (73.6)	41 (59.4)		
PSS, M (SD)	13.55 (6.76)	16.35 (5.10)	t = -3.15	0.002
PSS level, n (%)				0.023^a^
Low	57 (51.8)	22 (31.9)		
Moderate	51 (46.4)	45 (65.2)		
High	2 (1.8)	2 (2.9)		

PSS, perceived stress scale; M: mean; SD, standard deviation.

^a^ *p*-value was calculated using Fisher’s exact test.

### Perceived stress level

The average PSS score of the entire sample was 14.63 (SD = 6.31), and 44.1%, 53.6%, and 2.2% of the students were identified as having low, moderate, and high levels of COVID-19-related stress, respectively. As shown in Table 1, an independent-samples t-test revealed that the mean PSS score was significantly lower in third-year students than in fourth-year students (p = 0.002); third-year students’ average score was classified as low-stress level, whereas fourth-year students’ average score was classified as moderate-stress level. In line with this result, the distribution of the perceived stress levels also differed between third-year and fourth-year students (p = 0.023), with the majority of third-year students classified as having low stress and the majority of fourth-year students classified as having moderate stress.

When analyzed based on sex, neither the score (p = 0.168) nor the stress level (p = 0.589) differed significantly (S1 Table). Furthermore, there were no significant differences in PSS scores among the groups of students who chose different options for appropriate scope and participation levels for clinical clerkships, as determined by the one-way analysis of variance (scope: p = 0.424; participation: p = 0.052) (S2 Table).

### Students’ perception of appropriate clinical clerkship scope and participation during the COVID-19 pandemic

In the total sample, 70.9% (127/179) of students preferred that regular clinical rotations should have proceeded, and 64.2% (115/179) of students opted for active engagement in clerkship, despite the pandemic’s impact. Most of the third-year students (51.8%) chose a regular rotation schedule (except for ER, ICU, and isolation rooms), considering it to be the appropriate intra-pandemic scope of clerkship. Among the fourth-year students, the options of regular clinical rotations (except for ER/ICU/isolation rooms) and conducting rotations in general ward and outpatient clinic settings in a limited manner were the two most frequent responses (42.0% for each). Regarding appropriate participation levels for clerkship, third-year students most frequently responded that they would actively participate in clerkship activities and COVID-19-related volunteer work (38.2%). In contrast, fourth-year students chose participating in clerkship activities—but not in volunteer work—most frequently (39.1%). The detailed results are shown in Table 2.

**Table 2:** Fisher’s exact test for associations between medical students’ study year and clerkship-related perceptions during the COVID-19 pandemic.

	Total sample (n = 179)	Third-year students (n = 110)	Fourth-year students (n = 69)	*p*-value
Scope of clinical clerkship during the pandemic, n (%)				0.005
Proceed with regular rotation schedule (including ER/ICU).	41 (22.9)	30 (27.3)	11 (15.9)	
Proceed with regular rotation schedule (except for ER/ICU/isolation rooms).	86 (48.0)	57 (51.8)	29 (42.0)	
Carry out the clinical rotations in the general ward and outpatient clinic settings in small groups in a limited manner.	50 (27.9)	21 (19.1)	29 (42.0)	
Completely discontinue of all clinical rotations.	2 (1.1)	2 (1.8)	0 (0.0)	
Level of participation in clinical clerkship during the pandemic, n (%)				0.007
Actively participate in clinical rotation and COVID-19 pandemic-related volunteer work.	53 (29.6)	42 (38.2)	11 (15.9)	
Actively participate in clinical rotation but not in COVID-19 pandemic-related work.	62 (34.6)	35 (31.8)	27 (39.1)	
Participate only in essential clinical rotation for student safety (infection prevention).	50 (27.9)	28 (25.5)	22 (31.9)	
Minimize clinical rotations despite missing out on essential clinical rotations because students’ safety is the highest priority.	9 (5.0)	4 (3.6)	5 (7.2)	
Minimize clinical rotations, as students may be asymptomatic carriers.	5 (2.8)	1 (0.9)	4 (5.8)	

ER, emergency room; ICU, intensive care unit.

We also analyzed whether students’ study year was associated with clerkship-related perception options. The distribution of the students’ responses to the appropriate scope (p = 0.005) and the level of participation (p = 0.007) differed significantly across the groups (Table 2). On the other hand, no significant relationship was found between sex and clerkship scope (p = 0.145) or the level of participation (p = 0.405) (S3 Table).

### Factors related to preferences for clerkship scope and participation

A multinomial logistic regression was conducted to model whether the perception toward clerkship could be predicted based on the PSS score, sex, and study year. As the last options of clerkship-related variables were rarely selected by students, we have combined the last two options of each variable into one category for analysis. The first options for each served as reference categories; this presents a broader spectrum of clerkship and a higher level of participation in clinical rotations. For the appropriate clerkship scope, the addition of predictors improved the model compared to the intercept-only model (χ^2^ = 14.07, p = 0.029). As shown in Table 3, students were less likely to choose a limited-scope clerkship than a regular rotation schedule if they were third-year students rather than the fourth-year students. Both the stress level and sex were not statistically significant. Similarly, the full model significantly predicted the level of clerkship participation better than the intercept-only model (χ^2^ = 19.78, p = 0.019). Third-year students were less likely to select all types of limited clerkship participation compared to fourth-year students. The PSS score did not significantly predict the choice of clerkship participation level, but only for the most minimal clinical rotations compared to the reference category. The detailed results are shown in Table 4.

**Table 3:** Multinomial logistic regression model for associations of PSS scores, sex, and study year with the perception of appropriate clerkship scope during the COVID-19 pandemic.

	*B*	SE	Odds ratio (95% CI)	*p*-value
Category 1 vs. 2^a^				
PSS	0.010	0.030	1.01 (0.95–1.07)	0.741
Sex				
Female	0.645	0.461	1.91 (0.77–4.71)	0.162
Male (reference)	–	–	–	–
Year of study				
Third year	-0.225	0.434	0.80 (0.34–1.87)	0.603
Fourth year (reference)	–	–	–	–
Category 1 vs. 3^a^				
PSS	0.033	0.036	1.03 (0.96–1.11)	0.354
Sex				
Female	0.856	0.497	2.35 (0.89–6.23)	0.085
Male (reference)	–	–	–	–
Year of study				
Third year	-1.054	0.465	0.35 (0.14–0.87)	0.023
Fourth year (reference)	–	–	–	–

SE, standard error; CI, confidence interval; PSS, perceived stress scale.

^a^Category 1 (reference category): Proceed with regular rotation schedule (including ER/ICU); Category 2: Proceed with regular rotation schedule (except for ER/ICU/isolation rooms); Category 3: Combination of two options—1) Carry out the clinical rotations in the general ward and outpatient clinic settings in small groups in a limited manner, 2) Completely discontinue of all clinical rotations.

**Table 4:** Multinomial logistic regression model for associations of PSS scores, sex, and study year with the perception of appropriate clerkship participation level during the COVID-19 pandemic.

	*B*	SE	Odds ratio (95% CI)	p-value
Category 1 vs. 2^a^				
PSS	0.047	0.031	1.05 (0.99–1.11)	0.135
Sex				
Female	0.336	0.429	1.40 (0.60–3.24)	0.433
Male (reference)	–	–	–	–
Year of study				
Third year	-0.918	0.436	0.40 (0.17–0.94)	0.035
Fourth year (reference)	–	–	–	–
Category 1 vs. 3^a^				
PSS	0.031	0.033	1.03 (0.97–1.10)	0.341
Sex				
Female	0.278	0.450	1.32 (0.55–3.19)	0.538
Male (reference)	–	–	–	–
Year of study				
Third year	-0.977	0.454	0.38 (0.15–0.92)	0.032
Fourth year (reference)	–	–	–	–
Category 1 vs. 4^a^				
PSS	0.126	0.056	1.13 (1.02–1.27)	0.024
Sex				
Female	0.230	0.671	1.26 (0.34–4.69)	0.732
Male (reference)	–	–	–	–
Year of study				
Third year	-1.675	0.667	0.19 (0.05–0.69)	0.012
Fourth year (reference)	–	–	–	–

SE, standard error; CI, confidence interval; PSS, perceived stress scale

^a^Category 1 (reference category): Actively participate in clinical rotations and COVID-19 pandemic-related volunteer work; Category 2: Actively participate in clinical rotations but not in COVID-19 pandemic-related work; Category 3: Participate only in essential clinical rotations for student safety (infection prevention); Category 4: Combination of two options—1) Minimize clinical rotations despite missing out on essential clinical rotations because students’ safety is the highest priority, 2) Minimize clinical rotations, as students may be asymptomatic carriers.

## Discussion

This study investigated medical students’ intra-pandemic distress and perceptions toward clinical clerkship, particularly those in the clinical phase. Pandemic-related stress and clerkship-related perceptions differed significantly based on the students’ study year. Overall, third-year students experienced lower distress and showed more positive perceptions of clerkship participation compared to fourth-year students.

In the entire sample, the mean PSS score was 14.6, and more than half of the medical students were identified as having a certain level of COVID-19-related stress, among which about 2% of students were experiencing high levels of stress. Previous research findings involving the same instrument have shown that medical or health profession students’ perceived stress during the COVID-19 pandemic ranged from 17.8 to 20.4 [20–22]. Based on the average PSS score, the students in our study seem to experience relatively lower stress than those in previous studies. However, direct comparisons may be limited, as prior research enrolled students from all academic years, and the timings of investigations differed. A comparison between third- and fourth-year students showed that third-year students experienced significantly lower stress than fourth-year students. Since fourth-year students had undergone clinical rotations (and third-year students had not) when the survey was conducted, a possible explanation may be that this finding reflects the difference in distress levels between clinical and preclinical students. Previous studies conducted before the COVID-19 pandemic had reported that students in clinical years experienced more stress, burnout, and depressive symptoms compared to preclinical students [23, 24]. In addition to this preexisting gap, the pandemic may have imposed different loads on preclinical and clinical students in terms of learning and psychological distress [25, 26]. As the majority of the preclinical curriculum is based on didactic courses, which could have been delivered online, preclinical students may have experienced less disruption of education compared to clinical students and, thus, relatively little pandemic-related uncertainty [27, 28]. One unanticipated finding related to stress was that the PSS scores did not differ based on choices regarding the appropriate scope of clinical clerkship or level of participation in clinical activities, which echoed the finding of Compton *et al*. (2020) that scores for burnout did not differ based on the preference to return to the clinical setting during the early phase of the COVID-19 outbreak. The multinomial logistic regression result aligned with this finding as the PSS scores did not predict the preferences for limited clerkship scope or engagement, except for choosing the most minimized engagement against the most active engagement. These findings altogether suggest that pandemic-related stress seems to have little impact on clerkship-related perceptions in general, while there remains a possibility that higher stress may lead to inclinations toward minimal clerkship participation during the pandemic.

We have identified that more than half of the medical students in the total sample opted for regular clinical rotations and active participation in clerkship activities. In particular, third-year students entering the clinical phase were more likely to prefer clinical rotations involving high-risk departments for COVID-19 exposure, such as the ER and ICU, compared to fourth-year students, who had one year of prior clinical training. Similarly, third-year students preferred to be more involved in clinical activities, with most of them opting to volunteer in work related to the COVID-19 pandemic. In contrast, a higher proportion of fourth-year students chose to minimize clerkship participation compared to third-year students. These results correspond with the multinomial logistic regression finding that third-year students had lower odds of preferring a narrower scope for clerkship sites and more limited participation. Our finding was consistent with that of Kim *et al*. (2020), who also reported that third-year students were more likely to participate in clinical clerkship during the COVID-19 pandemic than fourth-year students in South Korea [15]. However, these results differed from another previous finding from an institution in Singapore that second-year students, who were in the first clinical clerkship year like our students, were less willing to prefer to return to the clinical setting than more senior-level students [16]. This discrepancy may be attributed to the similarities and differences in the educational and COVID-19-related context in previous studies; since Kim *et al*.’s study was conducted in South Korea [15], the structure of educational phases and the context and understanding of the COVID-19 outbreak situation may have been similar to our study. In South Korea, medical students generally engage in core clinical clerkships for the first time in their third year, and the curriculum for this phase usually consists of internal medicine, surgery, pediatrics, obstetrics and gynecology, psychiatry, and neurology. Although this core clerkship would require high levels of workload and intense contact with patients, students may have opportunities to immerse themselves within clinical settings and acquire confidence and identity of becoming a doctor [29]. Although Kim *et al*.’s study and this study have a 1-year difference in the investigation timing, there is a similarity in terms of the academic calendar timing in that both surveys were conducted at the beginning of a new academic year. The third-year students starting their clinical clerkship may have been more motivated to pursue a broader clinical experience and active engagement in a new educational setting. Meanwhile, since fourth-year students in our study had gained more practical experiences about the impact of COVID-19 at the time of the survey, they may have encountered more clerkship challenges, thus growing to prefer more limited participation and range. However, these results should be interpreted with caution, as we did not investigate other personal and environmental factors that could have affected the students’ preferences for clerkship participation. A greater focus on factors that influence the students’ perception and morale, such as autonomous motivation, a higher sense of professional responsibility, and altruism [16, 30], would be critical in providing essential clinical education of high quality during the COVID-19 pandemic.

This study had one major strength in that the options for breadth and depth of clinical clerkship were specifically described, which helped to build a concrete understanding of students’ preferences. Furthermore, as the study participants experienced an entire academic year under the influence of the pandemic, observing the waxing and waning of the situation, the study results may reflect stable preferences on this issue. However, despite the study’s strengths, there are several limitations that should be considered. The most important limitation lies in the fact that this study did not include other factors that could have affected the students’ distress and preference for clerkship activities and participation [12, 15], which limits our understanding as to why individual students prefer certain choices regarding clinical rotations. Thus, caveats should be noted that the rationale for interpreting the differences between third- and fourth-year students could be limited. As reported in a previous study, factors other than educational experiences, such as institutional strategy and support and whether students are aware of these, may also influence distress and apprehension related to the pandemic [12]. Second, the fourth-year students’ response rates were relatively low compared to that of the third-year students. Thus, the results may not adequately represent the viewpoint of the group. Third, there were only a few responses to several categories of survey items, which may have limited the validity of the statistical analysis. In this regard, we used Fisher’s exact test instead of the chi-squared test when necessary. In multinomial logistic regression, the last two options of each clerkship-related variable were combined into one category. However, this caveat should be noted in interpreting the results. Fourth, due to a cross-sectional design, we could not discern whether the differences between the third- and fourth-year students originated from the distinct cohort characteristics or the study year effect. Fifth, the generalizability of the results could be limited, as the study investigated students at only one medical school.

## Conclusions

Medical students’ intra-pandemic perceived stress and perceptions toward clinical clerkship differed based on study year. Third-year students, who were starting their clinical clerkship year, reported lower stress and were more inclined to engage in clerkship activities compared to fourth-year students who had experienced prior clerkship. Given the importance of maintaining adequate quantity and quality of clinical education, it is important to critically reflect on educational experiences during the pandemic. Further studies that focus on the qualitative aspects of students’ clerkship experiences could help to discern the differential impacts of key factors and improve the clinical learning environment at a time of significant change. In addition, the development of a new curriculum and educational strategies that are suited for intra-pandemic changes in the clinical environment during the pandemic should be considered [31, 32].

## Supporting information

S1 Table Perceived stress scale scores and stress severity levels by sex. (DOCX)

S2 Table Analysis of variance test results for comparisons of perceived stress scale scores by clerkship-related perception categories. (DOCX)

S3 Table Fisher’s exact test for associations between medical students’ year of study and clerkship-related perceptions during the COVID-19 pandemic. (DOCX)

S1 File Dataset. (XLSX)
