# Supplementary material for: Medical students’ perceived stress and perceptions regarding clinical clerkship during the COVID-19 pandemic
Source: PLoS One. 2022 Oct 31;17(10):e0277059. doi: 10.1371/journal.pone.0277059 (PMC9621432; doi:10.1371/journal.pone.0277059)
Supplement: S1 Table — (DOCX) [file pone.0277059.s001.docx]

Table S1. Perceived stress scale scores and stress severity levels by sex

|  | Male (n=122) | Female (n=57) | *t* statistic | *p*-value |
| --- | --- | --- | --- | --- |
| PSS, M (SD) | 14.18 (6.60) | 15.58 (5.55) | t=-1.39 | 0.168 |
| PSS level, n (%) |  |  |  | 0.589^a^ |
| Low | 57 (46.7) | 22 (38.6) |  |  |
| Moderate | 62 (50.8) | 34 (59.6) |  |  |
| High | 3 (2.5) | 1 (1.8) |  |  |

PSS, perceived stress scale; M: mean; SD, standard deviation.

^a^ *p*-value was calculated using Fisher’s exact test.
