# Supplementary material for: Medical students’ perceived stress and perceptions regarding clinical clerkship during the COVID-19 pandemic
Source: PLoS One. 2022 Oct 31;17(10):e0277059. doi: 10.1371/journal.pone.0277059 (PMC9621432; doi:10.1371/journal.pone.0277059)
Supplement: S2 Table — (DOCX) [file pone.0277059.s002.docx]

Table S2. Analysis of variance test results for comparisons of perceived stress scale scores by clerkship-related perception categories

|  | PSS scores, M (SD) | *F* statistic | *p*-value |
| --- | --- | --- | --- |
| Scope of clinical clerkship during the pandemic: |  | 0.938 | 0.424 |
| Proceed with regular rotation schedule (including ER/ICU). | 13.66 (7.17) |  |  |
| Proceed with regular rotation schedule (except for ER/ICU/isolation rooms). | 14.40 (6.20) |  |  |
| Carry out the clinical rotations in the general ward and outpatient clinic settings in small groups in a limited manner. | 15.74 (5.64) |  |  |
| Completely discontinue of all clinical rotations. | 16.50 (9.19) |  |  |
| Level of participation in clinical clerkship during the pandemic: |  | 2.402 | 0.052 |
| Actively participate in clinical rotation and COVID-19 pandemic-related volunteer work. | 12.85 (6.74) |  |  |
| Actively participate in clinical rotation but not in COVID-19 pandemic-related work. | 15.27 (5.73) |  |  |
| Participate only in essential clinical rotation for student safety (infection prevention). | 14.72 (6.12) |  |  |
| Minimize clinical rotations despite missing out on essential clinical rotations because students’ safety is the highest priority. | 17.78 (7.24) |  |  |
| Minimize clinical rotations, as students may be asymptomatic carriers. | 18.80 (4.60) |  |  |

PSS, perceived stress scale; M: mean; SD, standard deviation.
