# Supplementary material for: Medical students’ perceived stress and perceptions regarding clinical clerkship during the COVID-19 pandemic
Source: PLoS One. 2022 Oct 31;17(10):e0277059. doi: 10.1371/journal.pone.0277059 (PMC9621432; doi:10.1371/journal.pone.0277059)
Supplement: S3 Table — (DOCX) [file pone.0277059.s003.docx]

Table S3. Fisher’s exact test for associations between medical students’ year of study and clerkship-related perceptions during the COVID-19 pandemic

|  | Total sample (n=179) | Male (n=122) | Female (n=57) | *p*-value |
| --- | --- | --- | --- | --- |
| Scope of clinical clerkship during the pandemic, n (%) |  |  |  | 0.145 |
| Proceed with regular rotation schedule (including ER/ICU). | 41 (22.9) | 33 (27.0) | 8 (14.0) |  |
| Proceed with regular rotation schedule (except for ER/ICU/isolation rooms). | 86 (48.0) | 58 (47.5) | 28 (49.1) |  |
| Carry out the clinical rotations in the general ward and outpatient clinic settings in small groups in a limited manner. | 50 (27.9) | 30 (24.6) | 20 (35.1) |  |
| Completely discontinue of all clinical rotations. | 2 (1.1) | 1 (0.8) | 1 (1.8) |  |
| Level of participation in clinical clerkship during the pandemic, n (%) |  |  |  | 0.405 |
| Actively participate in clinical rotation and COVID-19 pandemic-related volunteer work. | 53 (29.6) | 40 (32.8) | 13 (22.8) |  |
| Actively participate in clinical rotation but not in COVID-19 pandemic-related work. | 62 (34.6) | 40 (32.8) | 22 (38.6) |  |
| Participate only in essential clinical rotation for student safety (infection prevention). | 50 (27.9) | 33 (27.0) | 17 (29.8) |  |
| Minimize clinical rotations despite missing out on essential clinical rotations because students’ safety is the highest priority. | 9 (5.0) | 7 (5.7) | 2 (3.5) |  |
| Minimize clinical rotations, as students may be asymptomatic carriers. | 5 (2.8) | 2 (1.6) | 3 (5.3) |  |

ER, emergency room; ICU, intensive care unit.
